# Anterolateral upper cervical approach for ventral C1–C2 meningioma

**DOI:** 10.3171/2023.6.FOCVID2377

**Published:** 2023-10-01

**Authors:** Serdar Rahmanov, Yücel Doğruel, Abuzer Güngör, Uğur Türe

**Affiliations:** 1 Department of Neurosurgery, Yeditepe University School of Medicine, Istanbul; and; 2 Department of Neurosurgery, University of Health Sciences, Bakirkoy Prof. Dr. Mazhar Osman Training and Research Hospital for Neurology, Neurosurgery and Psychiatry, Istanbul, Turkey

**Keywords:** C1–2 meningioma, anterolateral approach, upper cervical tumors

## Abstract

The surgical management of ventral upper cervical meningiomas poses significant challenges due to their deep location and limited accessibility. These tumors have the potential to compress or involve nearby neurovascular structures, leading to various neurological complications including inferior cranial nerve palsy, motor deficits, and sensory disturbances such as hypoesthesia, paresthesia, and impaired coordination. To address this issue, surgical intervention through an anterolateral or far lateral approach has been recognized as the optimal treatment strategy. In this video, the authors present a detailed demonstration of the operative technique using an anterolateral upper cervical approach to safely resect a ventrally located C1–2 meningioma.

## Transcript

This case demonstrates the anterolateral upper cervical approach for a ventral C1–2 meningioma.

### 0:28 Clinical Presentation.

A 59-year-old female patient presented to our institution with a 3-year history of paresthesia in both extremities. The patient had no neurological deficits.

### 0:44 Neuroimaging Findings.

Subsequent MRI imaging identified a ventrally located meningioma at the C1–2 level, resulting in compression of the spinal cord. Surgical removal was recommended. The anterolateral upper cervical approach was chosen to control the contralateral extension of the tumor.

### 1:00 Surgical Plan and Positioning.

Because of possible tumor adhesions to surrounding tissue, the anterolateral upper cervical approach was chosen instead of the posterior midline approach, especially to manage contralateral tumor extension.^1–3^ The patient is placed in the semilateral position with the head rotated 45° to the contralateral side. Critical equipment utilized includes intraoperative neurophysiological monitoring, neuronavigation, Doppler ultrasound, and the Cavitron ultrasonic surgical aspirator (CUSA).

### 1:55 Operative Techniques.

The skin incision begins anterior to the sternocleidomastoid muscle. It extends from the C4 level along the mastoid process to the level of the external auditory meatus, where it curves dorsally for 3 to 4 cm.^2,4,5^

After the skin incision, the sternocleidomastoid muscle together with suboccipital deep muscle group are separated from the mastoid and occipital bones, exposing the suboccipital area. The musculocutaneous flap is reflected dorsolaterally.

After exposing the lateral suboccipital area, the C1 transverse process is identified via palpation, and the V3 segment of the vertebral artery is identified with Doppler ultrasound. The great auricular nerve is dissected from the surrounding tissue and released.

At the next step, the spinal accessory nerve is identified and dissected. The transverse process of C1 is exposed through dissection of the superior and inferior obliquus capitis muscles. Before these muscles are dissected, it is essential to determine the exact location of the tip of the C1 transverse process and the horizontal portion of the V3 segment of the vertebral artery to prevent injury.^2,4,5^ The dissection is extended to expose the posterior arch of the C1 and the V3 segment of the vertebral artery. The horizontal portion of the V3 segment of the vertebral artery is dissected and the atlanto-occipital ligament is exposed. The V2 segment of the vertebral artery is identified with Doppler ultrasound. The jugular vein is then exposed and retracted away.

In this view, we observe the dorsal ramus of the C2 nerve. To gain access to the lamina of C2, the dorsal ramus of the C2 nerve is sacrificed, allowing better exposure and visualization of the targeted area. Here intraoperative photography shows the anatomical landmarks and the surgical corridor. The posterior atlanto-occipital ligament is incised and retracted to expose the dura. Then, a left-sided unilateral removal of the posterior arch of C1 and hemilaminectomy of C2 are completed.

This image demonstrates the surgical exposure and surrounding anatomical structures. A linear incision is made along the dura and CSF is released. The arachnoid membrane overlying the tumor and spinal cord is sharply dissected. The tumor is located ventral to the spinal cord and dentate ligament. The roots of the C2 and C3 nerves and the spinal accessory nerve are identified. The dentate ligaments are cut to expose the tumor and mobilize the spinal cord and tumor.

### 6:44 Tumor Removal.

Some samples are taken from the tumor and collected for histopathological examination. The central part of the tumor is debulked with the CUSA. After the tumor capsule collapses, the dural attachment of the tumor is cut. The tumor tissue was soft and not attached to the spinal cord. This allowed the tumor to be removed in one piece.

Here we see the anastomosis roots from C2 to C3. Between these roots, the residual tumor is seen at its origin and removed. Once the tumor is removed, the dural origin of the tumor is coagulated, scraped, and removed along with the surrounding arachnoid membrane.

### 8:21 Closure.

A thin layer of gelatin sponge is placed between the dura and spinal cord to prevent postoperative meningospinal adhesions. The dura is tightly sutured with autograft muscle to prevent CSF leakage. To prevent postoperative adhesions, the gelatin sponge is positioned over the dura mater and the vertebral artery. The block of musculocutaneous flap is sutured tightly to the nuchal line, and the wound is closed.

### 9:13 Outcome.

Early postoperative MRI imaging confirmed complete removal of the tumor. Histopathological examination revealed a WHO grade 1 angiomatous meningioma. The postoperative period was uneventful.

Thank you.

## Disclosures

The authors report no conflict of interest concerning the materials or methods used in this study or the findings specified in this publication.

## Author Contributions

Primary surgeon: Türe. Editing and drafting the video and abstract: all authors. Critically revising the work: all authors. Reviewed submitted version of the work: all authors. Approved the final version of the work on behalf of all authors: Türe. Supervision: Türe.

## Supplemental Information

### Patient Informed Consent

The necessary patient informed consent was obtained in this study.
